# Mechanism of Human Papillomavirus Binding to Human Spermatozoa and Fertilizing Ability of Infected Spermatozoa

**DOI:** 10.1371/journal.pone.0015036

**Published:** 2011-03-07

**Authors:** Carlo Foresta, Cristina Patassini, Alessandro Bertoldo, Massimo Menegazzo, Felice Francavilla, Luisa Barzon, Alberto Ferlin

**Affiliations:** 1 Section of Clinical Pathology and Centre for Male Gamete Cryopreservation, Department of Histology, Microbiology and Medical Biotechnologies, University of Padova, Padova, Italy; 2 Andrology Unit, Department of Internal Medicine, University of L'Aquila, Coppito (L'Aquila), Italy; 3 Section of Microbiology and Virology, Department of Histology, Microbiology and Medical Biotechnologies, University of Padova, Padova, Italy; University of Liverpool, United Kingdom

## Abstract

Human papillomaviruses (HPVs) are agents of the most common sexually transmitted diseases in females and males. Precise data about the presence, mechanism of infection and clinical significance of HPV in the male reproductive tract and especially in sperm are not available. Here we show that HPV can infect human sperm, it localizes at the equatorial region of sperm head through interaction between the HPV capsid protein L1 and syndecan-1. Sperm transfected with HPV E6/E7 genes and sperm exposed to HPV L1 capsid protein are capable to penetrate the oocyte and transfer the virus into oocytes, in which viral genes are then activated and transcribed. These data show that sperm might function as vectors for HPV transfer into the oocytes, and open new perspectives on the role of HPV infection in males and are particularly intriguing in relation to assisted reproduction techniques.

## Introduction

Human papillomaviruses (HPVs) comprise a highly diverse group of small, non-enveloped double-stranded DNA viruses that belong to the *Papillomaviridae* family [1], [2]. They are agents of the most common sexually transmitted diseases [3] that can infect both females and males. HPVs commonly infect mucosal genital epithelia, with an estimated 75% of humans being affected during life [4], [5]. HPV infection is very common among men and women across all geographical, racial and socio-economic subgroup worldwide. More than 100 types of HPV have been identified and about 40 types infect the anogenital region. Anogenital HPV types have been further classified into low-risk, which are associated with anogenital warts and mild dysplasia, and high-risk types, which are associated with high-grade dysplasia and anogenital cancers, such as cervical and anal carcinoma [3], [6].

In males, HPV DNA and RNA have been well documented not only in the anal region, perineal area, scrotum, glans, penile shaft, and urethra [6], [7], but also in the reproductive system (testis, epididymis, and ductus deferens) [7]–[10]. Moreover, several reports documented the presence of HPV in the semen [7], [11], [12]. We have previously described a 10% prevalence of semen HPV infection in asymptomatic sexually active young adult men, showing that HPV can be localized in the sperm head and that infected spermatozoa had a significant reduction in mean sperm motility [13]. Moreover, we have recently demonstrated a higher prevalence of infection in the semen of patients with risk factors for HPV (patients with genital warts and partners of infected females) than in controls [14].

However, precise data about the presence and significance of HPV in sperm are not available. In particular, the exact localization and mechanism of infection by HPV in sperm, as well as the role of infected sperm cells as a transmission vector for the virus are still unknown. It is not known whether HPV-infected sperm are able to infect the partner and whether they are able to fertilize. This is also of crucial importance in relation to in vitro fertilization techniques because of the possibility that sperm infected with HPV injected in the oocyte cytoplasm (like during the intra-cytoplasmic sperm injection procedure) could interfere with fertilization, implantation, embryo development, premature abortion, and definitively with outcome and safety of assisted reproduction techniques (ART). Infertile patients seeking ART are at higher risk to carry HPV in sperm (10% with respect to 0% of fertile controls) [14] and HPV has been demonstrated in about 6% of sperm samples cryopreserved from testicular cancer patients [15], who need ICSI to become father.

In this study we aimed at better understand the mechanism of infection by HPV in human sperm and the ability of HPV-infected sperm to fertilize and transfer HPV DNA and capsid proteins to the oocyte. We used different in vitro methods to localize HPV DNA in sperm and to show the interaction between the capsid protein L1 and the primary attachment receptor syndecan-1 on sperm. Then, we used the hamster egg-human sperm penetration test to show the ability of sperm to transfer the capsid protein L1 to the oocyte and the transfer of E6/E7 viral genes to the oocyte by transfected sperm and the expression of these genes by the oocyte.

## Results and Discussion

**Figure 1:**
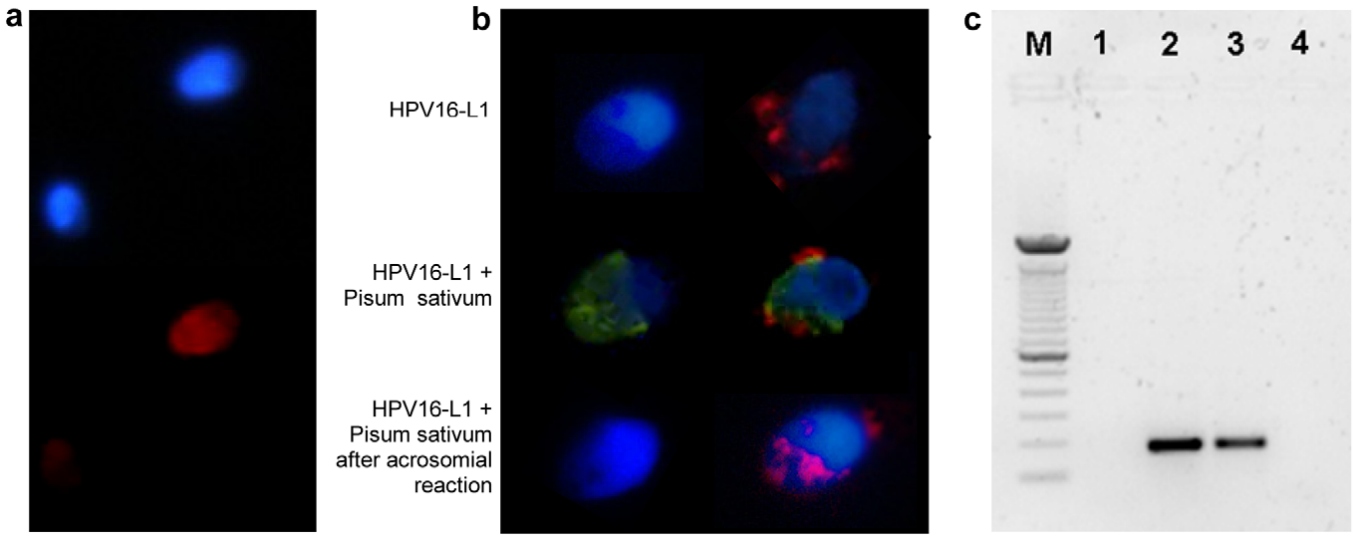
Detection and localization of HPV in human sperm. **a**. Fluorescence in situ hybridization (fluorescence microscope) for HPV DNA on sperm from a patient with HPV16 in semen. Infected and noninfected sperm are shown. Red: HPV DNA (Texas red); blue: nuclear staining (DAPI). **b**. Immunofluorescence (confocal fluorescence microscope) for HPV16 capsid protein L1 on sperm from a control (left) and a patient with HPV16 in semen (right). Upper panel, L1 antibody; central panel, L1 antibody and Pisum Sativum (acrosome); lower panel, L1 antibody and Pisum Sativum after induction of the acrosome reaction. Red: HPV16 L1; green: Pisum Sativum; blue: nuclear staining (DAPI). **c**. PCR for HPV E7 gene from sperm DNA. Lane M: DNA marker (100 bp); 1: negative control (no template); 2: positive control (sperm transfected with recombinant plasmid pIRES2-AcGFP1-E6E7); 3: sperm from a patient with HPV16 in semen; 4: sperm from a control subject.

Human sperm from a male previously found to carry HPV16 in semen [13] were studied by fluorescence in situ hybridization (FISH) using biotin-labeled HPV DNA probe (a mix of total genomes containing the conserved HPV region) and visualized by fluorescence microscope (Fig. 1a). A clear signal was found in about 25% of sperm and the fluorescence was evident in the sperm head. To confirm this analysis and better understand the precise localization in the sperm head, we performed immunofluorescence analysis using an antibody against the capsid protein L1 in intact sperm and sperm which underwent acrosome reaction (Fig. 1b). The signal for L1 was confirmed to be exclusively localized in the sperm head, especially at the equatorial region. After acrosome reaction the signal persisted in this region, excluding therefore the presence of HPV in the acrosome and suggesting a preferential localization of HPV in the equatorial region. PCR analysis for the L1 gene confirmed the infection of sperm by HPV (Fig. 1c). Taken together, these data show that sperm might be infected by HPV and that both HPV DNA and L1 capsid protein are found in the sperm head, almost exclusively in the equatorial region.

**Figure 2:**
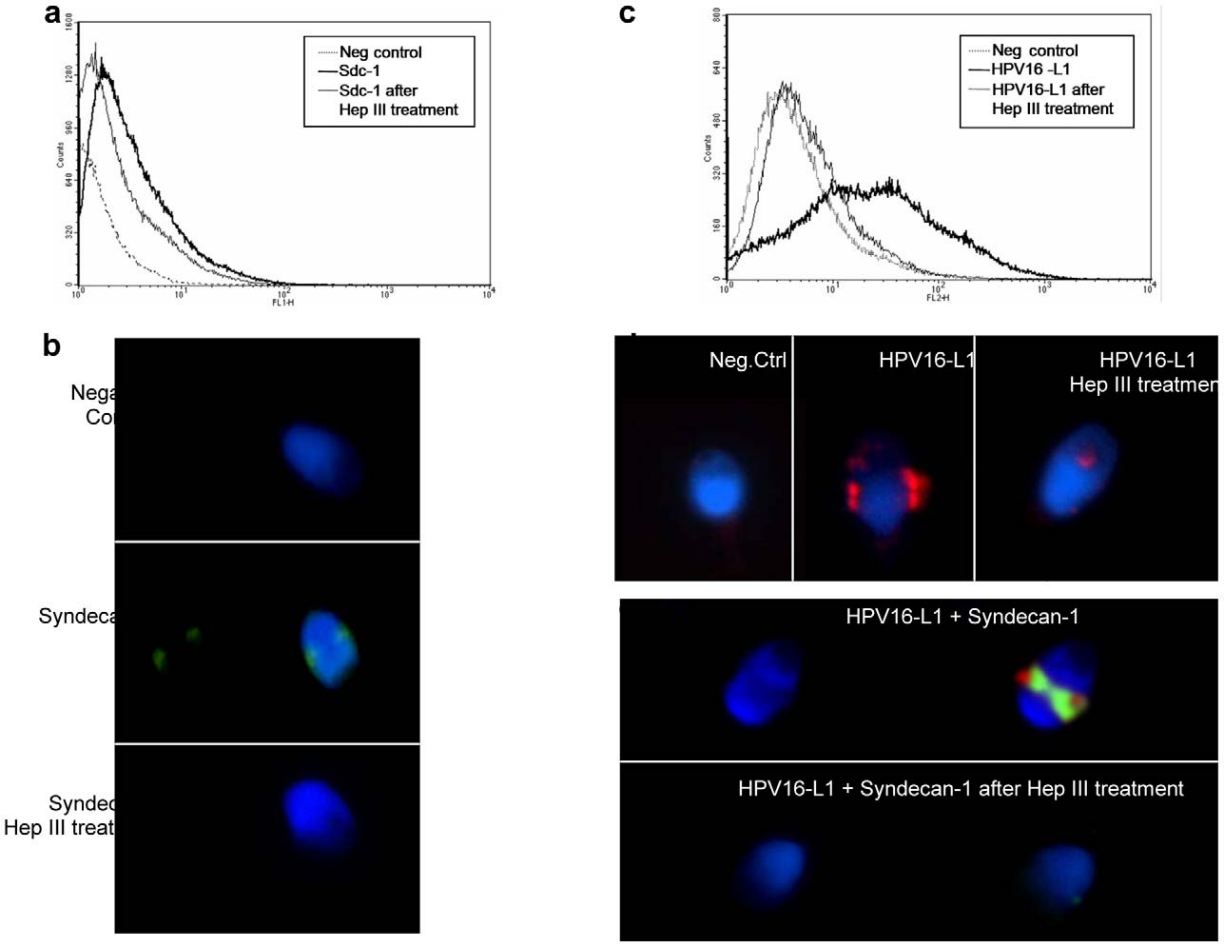
HPV interacts with syndecan-1 on human sperm. **a**. Cytofluorimetric analysis with mouse anti-human CD138 (syndecan-1), FITC conjugated on sperm before and after treatment with Heparinase-III. Samples without anti CD138 were used as negative controls. **b**. Immunofluorescence analysis (confocal fluorescence microscope) for syndecan-1 (CD138, FITC conjugated of sperm before and after treatment with Heparinase-III. Upper panel: negative control (omission of CD138); central panel: syndecan-1; lower panel: syndecan-1 after Heparinase-III treatment. **c**. Cytofluorimetric analysis of sperm exposed to HPV16 L1 capsid protein, treated or not with Heparinase-III and analyzed with mouse monoclonal HPV16 L1 antibody. Samples incubated with secondary phycoerythrin anti-mouse antibody alone were used as negative controls. **d**. Immunofluorescence analysis for HPV L1 capsid protein (confocal fluorescence microscope) in sperm exposed to HPV16 L1 treated or not with Heparinase-III and analyzed with mouse monoclonal antibody anti HPV16 L1 and DAPI. **e**. Colocalization analysis of HPV L1 capsid protein and syndecan-1 by immunofluorescence (confocal fluorescence microscope) of sperm exposed to HPV16 L1 treated or not with Heparinase-III and analyzed with mouse anti-human CD138 (syndecan-1) monoclonal antibody, FITC conjugated and mouse monoclonal antibody anti HPV16 L1 and DAPI.

Cell entry of HPV is initiated by binding of the virus particle to cell surface receptors, a prerequisite for subsequent interaction with the elusive uptake receptor, internalization and deliver of the genetic material into the nucleus of the target cell [16]–[19]. The HPV genome is surrounded by an icosahedral capsid composed by two structural proteins, the major protein L1 and the minor capsid protein L2 [18], [20]. Initial binding to the cell surface is mediated by L1 that interacts with heparan sulfate proteoglycans (HSPGs), followed by conformational changes and proteolytic cleavage of L2 that is essential for successful infection [16], [19]. Among HSPGs, syndecan-1 seems to represent the primary attachment receptor [16], [19], [21]–[25]. Syndecan-1 is a widely expressed membrane-bound proteoglycan found predominantly in the epithelial cells [26], but no information exists about its possible presence in sperm. To test the hypothesis that HPV infects sperm by binding with syndecan-1, we performed flow cytometric and immunofluorescence analyses of native sperm and sperm exposed to HPV16 capsid (Fig. 2). These analyses showed that sperm express syndecan-1 most exclusively in the equatorial region of sperm head (Fig. 2b) and both flow cytometry and immunofluorescence were blocked by the use of heparinase III (Fig. 2a and 2b), confirming the specificity of the reaction. To analyze whether HPV interacts with syndecan-1, sperm were exposed to HPV16 L1 capsid protein and then treated with heparinase III. Cytofluorimetric and immunofluorescence analyses showed that treatment with Heparinase III abolished the binding of HPV to sperm (Fig. 2c and 2d). Furthermore, immunofluorescence analysis showed that HPV16 L1 protein and syndecan-1 colocalize in the equatorial region of sperm head, and the binding was completely abolished by treatment of sperm with Heparinase III (Fig. 2e). These data clearly show that HPV infection of human sperm implies attachment of the HPV capsid protein L1 to syndecan-1 in the equatorial region of sperm head.

**Figure 3:**
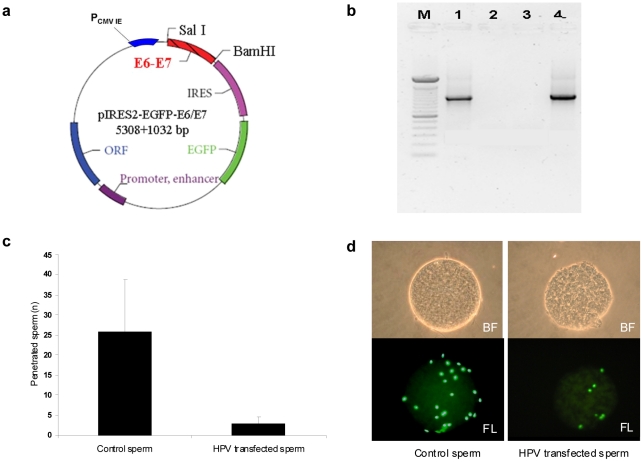
Sperm transfected with E6/E7 plasmid penetrate oocytes. **a**. Scheme of the recombinant plasmid pIRES2-AcGFP1-E6E7. E6/E7 genes have been amplified (1032 bp) from plasmid p1321 HPV-16 E6/E7 by PCR and subcloned to plasmid pIRES2-AcGFP1 between SalI and BamHI restriction sites. **b**. PCR for HPV E6/E7 genes from transfected sperm. Lane M: DNA marker (100 bp); 1: sperm transfected with recombinant E6/E7 plasmid; 2: negative control (no template); 3: sperm transfected only with Lipofectamine 2000; 4: positive control (pIRES2-AcGFP1-E6E7 plasmid). **c**. Mean number of human sperm penetrated per hamster oocyte in control and sperm transfected with HPV-16 E6/E7 plasmid. **d**. Hamster oocytes penetrated by control sperm and sperm transfected with HPV16 E6/E7 plasmid in bright field (BF, upper panel) and fluorescence (FL, lower panel) using SYBR green DNA stain.

**Figure 4:**
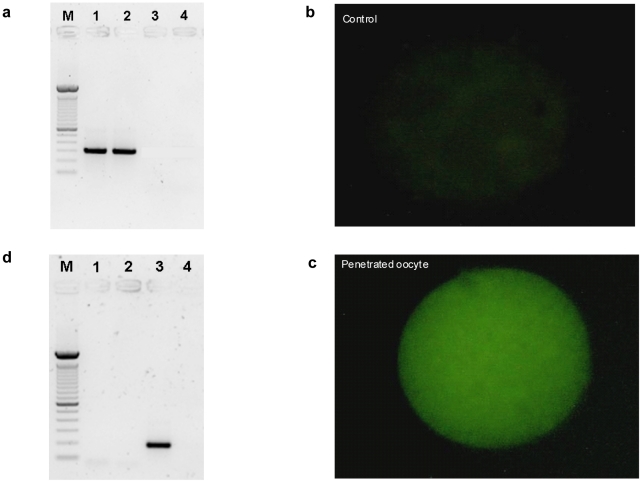
Oocytes penetrated by sperm transfected with HPV E6/E7 express HPV E6/E7 genes. **a**. PCR for E6 gene from single oocyte penetrated by transfected sperm. Lane M: DNA marker (100 bp); 1: positive control (pIRES2-AcGFP1-E6E7 plasmid); 2: single oocyte penetrated by sperm transfected with recombinant plasmid; 3: negative control (no template); 4: single oocyte penetrated by sperm transfected only with Lipofectamine 2000. **b**. GFP-E6 expression in oocyte penetrated by control sperm. **c**. GFP-E6 expression in oocyte penetrated by transfected sperm. **d**. RT-PCR for E6 gene from single oocyte penetrated by transfected sperm. Lane M: DNA markers (100 bp); 1: negative control (no template for Reverse Transcription); 2: negative control (no template for PCR); 3: single oocyte with green fluorescence penetrated by transfected sperm; 4: single oocyte without green fluorescence penetrated by control sperm.

**Figure 5:**
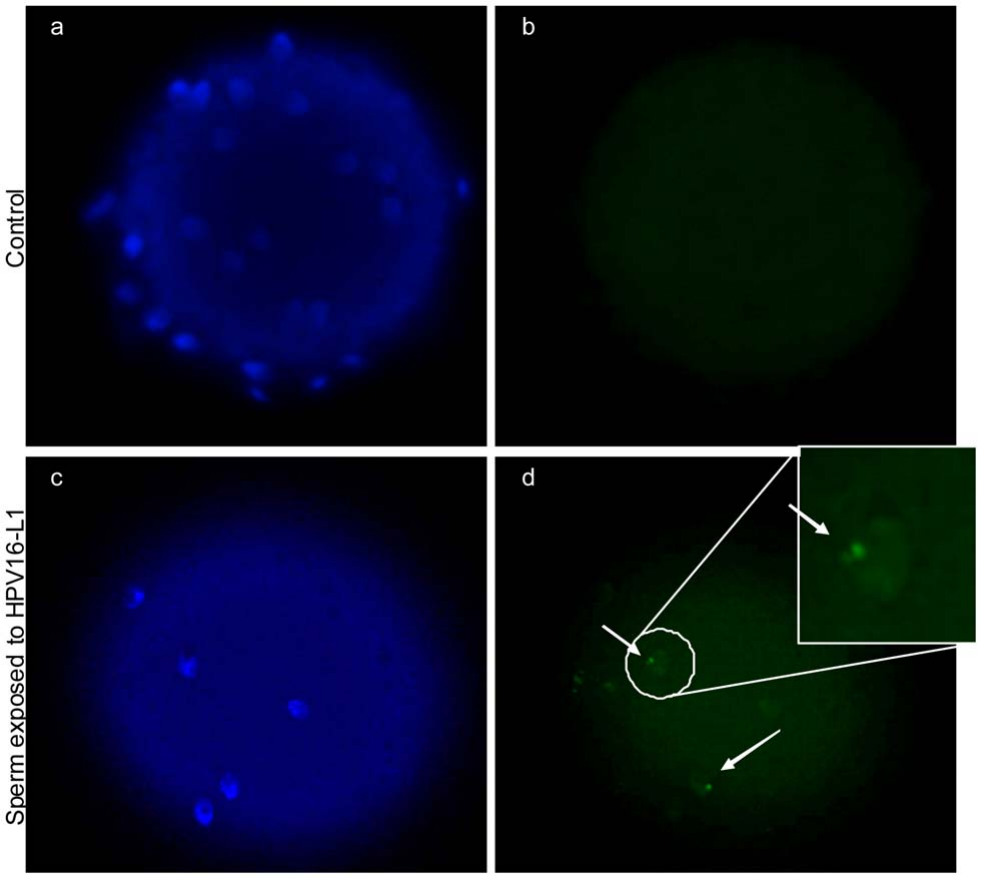
Transfer of HPV L1 capsid protein from human sperm to hamster oocytes. Immunofluorescence (confocal fluorescence microscope) for HPV16 L1 on hamster oocytes penetrated by human sperm exposed to HPV L1. Upper panel: oocytes penetrated by control sperm; lower panel: oocytes penetrated by sperm exposed to L1. Left; nuclear staining with DAPI (blue); right: HPV L1 antibody (green).

We then studied the fertilizing ability of HPV infected sperm and the possible transfer of the virus into oocytes. To this aim we used the hamster egg-human sperm penetration test (HEPT) using sperm transfected with a plasmid containing the HPV genes E6/E7 linked to GFP and using sperm exposed to the HPV capsid protein L1. The E6/E7 genes were amplified from plasmid p1321 HPV-16 E6/E7 and subcloned into plasmid pIRES-AcGFP1 to construct recombinant plasmid pIRES-AcGFP1-E6E7 (Fig. 3a), which was then used to transfect human sperm. After confirmation of successful transfection, HEPT demonstrated that human sperm transfected with HPV E6/E7 genes were able to penetrate hamster oocytes, even if the mean number of sperm penetrated per oocyte was lower than that obtained with control sperm (Fig. 3c). SYBR green staining for DNA confirmed the successful penetration of oocytes by transfected sperm (Fig. 3d). The successful penetration was confirmed by PCR analysis of E6 gene on single oocytes (Fig. 4a). Then, we analyzed whether oocytes penetrated by sperm transfected with HPV E6/E7 express the viral genes. Since the E6/E7 genes were linked to GFP in the plasmid, oocytes penetrated by transfected sperm had a green fluorescence if E6/E7 genes were transcribed. This analysis showed that oocytes express GFP when HPV transfected sperm penetrated (Fig. 4c), suggesting an active transcription of E6/E7 genes by the oocyte. This finding was confirmed by RT-PCR for the E6 gene performed on single oocytes with green fluorescence (Fig. 4d). Taken together these data showed that sperm transfected with E6/E7 genes are able to penetrate the oocyte, to deliver HPV genome in the oocyte, and that HPV genes are then actively transcribed by the penetrated oocyte. However, these data might not truly reflect the in vivo situation because the use of transfected sperm is obviously an artificial condition. Therefore, we performed HEPT with sperm exposed to HPV L1 capsid protein and we found that sperm are able to transfer the L1 protein into the oocyte (Fig. 5).

Taken together, the findings of the present study show that sperm might be infected by HPV and that the mechanism of entry of the virus in the sperm is mediated by interaction of the HPV L1 capsid protein with syndecan-1 that is mainly localized in the equatorial region of the sperm head. Other viruses found in association with sperm, such as HIV and HSV, have been shown to interact with HSPGs [27]–[31], suggesting this mechanism is a general strategy used by sexually transmitted viruses to infect sperm and overcome long distances and mucus barriers in the female genital tract. More interestingly, we were able to show that both sperm transfected with HPV E6/E7 genes and sperm exposed to HPV L1 capsid protein are capable to penetrate the oocyte, but this fertilization process seems to be impaired. Anyway, sperm might function as vectors for HPV transfer into the oocytes, as both L1 protein and E6/E7 “transforming” genes are delivered into the oocyte. E6/E7 genes once in the cytoplasm of the oocytes are then activated and transcribed.

These findings confirm previous studies that showed that HPV16 capsids bind at the equatorial region of the sperm head [32], but highlight for the first time the mechanism of interaction between HPV and sperm and the delivery and activation of HPV genome into the oocyte during natural fertilization. Previous studies [33], [34] showed that incubation with HPV is able to transfer viral DNA into sperm and that the infected cells can deliver exogenous DNA to the cumulus cells surrounding ovulated oocytes at the time of fertilization. It is not known whether there are risks for the partners of an HPV-infected male or for the embryos fertilized by infected sperm. Anyway, our data suggest that HPV-infected sperm have lower fertilizing ability, and these findings are particularly important during ART procedures. Furthermore, we recently demonstrated that HPV-infected sperm have reduced motility, and a possible role for HPV semen infection has been suggested for male infertility related to altered sperm motility (asthenozoospermia) [13], [14]. Although further studies are necessary to better understand the possible clinical implications of the findings of the present study, these data open new perspectives on the role of HPV infection in males.

## Methods

### Ethics Statement

The study was approved by the Institutional Ethics Committee of the Hospital of Padova and written informed consent was provided by participants.

### Semen sample collection and preparation

Human semen samples from volunteers were obtained by masturbation after 2–5 days of sexual abstinence in sterile containers. Samples were allowed to liquefy for 30 minutes at room temperature and semen volume, pH, sperm concentration, viability, motility, and morphology were determined following World Health Organization guidelines for semen analysis [35]. Sperm were separated by washing semen three times with Modified HTF medium with human serum albumin 5 mg/ml (Sperm washing medium, Irvine Scientific, Santa Ana, CA) and centrifuged at 300 g for 10 minutes. The pellet was used to collect the spermatozoa after swim-up technique (500 µl of sperm washing medium was layered gently up to the pellet, the tube was slanted to 45° and incubated for 1 h at 37°C in a 5% CO_2_ incubator), the collected spermatozoa were used for subsequent analyses. Subjects have been described before [13], and sperm were investigated for HPV identification and localization (HPV DNA, HPV16-L1 capsid), by fluorescence in situ hybridization, immunofluorescence and PCR as described below.

### Fluorescence *in Situ* Hybridization (FISH) for HPV in human sperm

Sperm samples containing at least 2×10^6^ sperm were fixed in a methanol-acetic acid solution for at least 1 hour at −20°C. To permeabilize sperm membrane, samples were treated with pepsin (1∶25,000, 0.01 mol/L) in prewarmed HCl 1 mol. for 10 minutes at 37°C. Permeabilization of the specimens was stopped with 4 min. washes in PBS 1×. Samples were then dehydrated in 70%, 80%, and absolute ethanol for 2 minutes and finally air-dried. Samples were then overlaid with 20 μL of hybridization solution containing biotin (BIO)-labeled HPV DNA probe (a mix of total genomes 7–8 Kb, containing the conserved HPV region) (Rembrandt in situ hybridization and detection kit HPV, Pan Path, Amsterdam, Netherlands). Each slide was covered with a glass coverslip and the edges were sealed with nail polish to prevent loss of the mixture during denaturation and hybridization. After denaturation of cellular target DNA and HPV DNA probe on a heating block for 5 min. at 95°C, hybridization was performed by incubating the samples at 37°C overnight in a humidified chamber. Thereafter, the coverslips were carefully removed and the slides were washed in PBS 1× for 10 minutes. After 15 minutes of incubation at 37°C with a wash solution to eliminate non-specific bound probe for use in situ hybridization (ISH) (R013R.0000 Pan Path, Amsterdam, Netherlands), the slides were washed three times in PBS 1× for 3 minutes. The negative control was processed in the same way, but omitting the viral probe. The biotin-labeled HPV probe was detected by incubation with 1∶200 streptavidin Texas red (Vector Laboratories, Burlingame, CA) for 40 minutes at room temperature. After detection, the slides were washed twice in PBS 1×/0.01% Triton and then twice in PBS 1× and mounted with a solution containing 4,6-diamino-2-phenylindole (DAPI 5 mg/mL) and antifade (BioBlue; BioView, Nes Ziona, Israel). Samples were analyzed using a fluorescence microscope (Nikon, Eclipse E600, Melville, NY, USA) equipped with a triple band-pass filter set (fluorescein isothiocyanate [FITC], tetrarhodamine isothiocyanate [TRITC], DAPI). For each slide 200 spermatozoa were analyzed. When nuclei were completely and homogeneously stained and when multiple small spots or single large signals were present, the sperm cells were classified as positive. The method was tested on control slides containing CaSki cells (R013R.0000 Pan Path, Amsterdam, Netherlands), human cervical carcinoma cell line with stably integrated and transcriptionally active HPV genomes that served as control for the specific probe.

### Immunofluorescence for HPV16-L1 capsid protein in human sperm

Sperm were resuspended at a concentration of 20×10^6^ sperm/mL in sperm washing medium and incubated with 20 µg/ml HPV16-L1 (Gardasil, Sanofi Pasteur MSD, Lyon, France) for 1 h at 37°C. The reaction volume was 300 µL. After incubation, motile spermatozoa were harvested by the swim-up technique as described above. The swim-up sperm fraction was divided in two samples used for later HPV16-L1 capsid detection after or not induction of acrosome reaction by calcium Ionophore III (Sigma-Aldrich) at 10 µM. Sperm without incubation with HPV16-L1 were used as negative control.

For immunofluorescence, 10 µl of sperm sample was smeared on clean, grease-free slides, air-dried, and fixed in PBS/paraformaldehyde 4% for 15 minutes. The glasses were washed three times in PBS for 5 minutes at room temperature and then used for the HPV capsid (HPV16-L1) and acrosome (pisum sativum) detection.

For HPV16-L1 detection the sperm were incubated with mouse monoclonal antibody HPV 16-L1 (CAMVIR-1) (0.8 µg/mL, 1∶250, Santacruz, Santa Cruz, CA) for 120 minutes at room temperature, and then washed in 0.2% tween-PBS for 5 minutes. Immunoreaction was detected by sequential incubation with biotinylated goat anti-mouse immunoglobulin secondary antibody (1∶200, Vector Laboratories, Burlingame, CA) and streptavidin Texas red (1∶400, Vector Laboratories, Burlingame, CA) both for 60 minutes at room temperature. After that the slides were incubated with the acrosome specific Pisum Sativum (1 µg/ml) for 30 minutes at room temperature and then washed twice in PBS. Nuclei were counterstained with DAPI 5 mg/mL, slides were mounted with anti-fade buffer and 24×24 mm coverslip. Immunostaining was evaluated with Nikon ViCo Video Confocal Microscope.

### HPV DNA (L1 gene) detection in human sperm

DNA extraction from swim-upped sperm was performed by QIAamp DNA mini kit (Qiagen, Valencia, CA). The presence of HPV DNA sequence (L1 gene) was investigated by nested polymerase chain reaction (PCR) using MY09/MY11 as outer primers and GP5þ/GP6þ as inner primers. The amplification products were analyzed by staining with SYBR safe DNA gel stain, after electrophoresis on 2% agarose gel. PCR amplification was carried also with a positive control (sperm transfected with recombinant plasmid pIRES2-AcGFP1-E6E7, see below) and a negative control (no template).

### Flow cytometry and immunofluorescence for CD138 (Syndecan-1) and HPV16-L1 capsid protein on human sperm

To evaluate the interaction between syndecan-1 and HPV16 capsid, fresh spermatozoa were washed twice in sperm washing medium and centrifuged (300 g for 10 minutes). The pellet was resuspended in PBS and divided into two aliquots with 40×10^6^ sperm cells with and without incubation with 3.5 U/ml Heparinase-III (Sigma Aldrich, St. Louis, MO) for 60 minutes at 37°C. Sperm were incubated with HPV16-L1 protein (20×10^6^ sperm/mL were resuspended in sperm washing medium and incubated with 20 µg/ml HPV16-L1 protein for 1 h at 37°C).

To detect CD138 (Syndecan-1) and HPV16-L1 in cytofluorimetry sperm were incubated with mouse anti-human CD138 monoclonal antibody, FITC conjugated, clone 1D4 (1∶5, Biolegend, San Diego, CA) or with mouse monoclonal antibody HPV16-L1 (CAMVIR-1) (1 µg/mL) (1∶200, Santacruz, Santa Cruz, CA) both on ice for 45 minutes in the dark. Sperm were washed twice in phosphate-buffered saline (PBS) and centrifuged at 300 g for 5 minutes. For HPV16-L1, the second layer phycoerythrin anti-mouse antibody (PE, 10 µM, Becton Dickinson, Franklin Lakes, NJ) was added to the cells and incubated for 45 minutes on ice. A multiple myeloma cell line U266 (ATCC, Manassas, VA, USA) were used as positive controls for syndecan-1, and the samples incubated with secondary antibody alone were used as negative controls for HPV16-L1. Samples were washed twice in PBS and centrifuged at 300 g for 5 minutes. The pellet was resuspended in 300 µl of PBS and then analyzed on a FACScalibur (Becton Dickinson, Oxford, UK) using Cellquest to acquire and analyze the data.

To detect CD138 (Syndecan-1) and HPV16-L1 colocalization in immunofluorescence, 10 µl of sperm samples were smeared on clean, grease-free slides, air-dried, and fixed in PBS/paraformaldehyde 4% for 15 minutes. The glasses were washed three times in PBS for 5 minutes, incubated with mouse anti-human CD138 monoclonal antibody, FITC conjugated, clone 1D4 (1∶10, Biolegend, San Diego, CA) overnight at 4°C, washed twice in PBS and incubated with mouse monoclonal antibody HPV 16-L1 (CAMVIR-1) (1∶250, 0.8 µg/mL, Santacruz, Santa Cruz, CA) for 120 minutes at room temperature. The slides were washed twice in 0.2% tween-PBS for 5 minutes. Immunoreaction for the HPV16-L1 was detected by sequential incubation with biotinylated goat anti-mouse immunoglobulin secondary antibody (1∶200 from Vector Laboratories, Burlingame, CA) and streptavidin Texas red (1∶400, Vector Laboratories, Burlingame, CA) both for 60 minutes at room temperature. The slides were washed twice in PBS, and nuclei were counterstained with (DAPI 5 mg/mL). Slides were mounted with anti-fade buffer and 24×24 mm coverslip. Immunostaining was evaluated with Nikon ViCo Video Confocal Microscope. Sperm without incubation with CD138 (Syndecan-1) and HPV16-L1 were used as negative control.

### Construction of recombinant plasmid pIRES2-AcGFP1-E6E7

The expression vector for HPV E6/E7 genes and GFP was constructed as Figure 3a. The E6/E7 genes were amplified (1032 bp) from plasmid p1321 HPV-16 E6/E7 (Clontech, Mountain View, CA) by PCR and subcloned into plasmid pIRES2-AcGFP1 (Clontech, Mountain View, CA) to construct recombinant plasmid pIRES2-AcGFP1-E6E7. The PCR mixture consisted of 5 µL Expand high fidelity buffer with MgCl_2_, 1 µL of PCR grade nucleotide mix (10×), 20 pmol of each primer with SalI and BamHI restriction sites (New England Biolabs, Ipswich, MA) including forward: 5′-GTGCGCGTCGACGTGATGCACCAAAAGAGAACTG-3′ and reverse: 5′-GTGCGCGGATCCGTGTGGTTTCTGAGAACAGATG-3′, 0,75 µL Expand high fidelity enzyme mix (Expand High Fidelity PCR System dNTPack, Roche Applied Science, Mannheim, Germany) and sterile H_2_O in a final volume of 50 μL. The amplification program was as follows: denaturing at 94°C for 2 minutes, 10 cycles each at 94°C for 15 seconds, annealing at 55°C for 30 seconds and extension at 72°C for 1 minute, 20 cycles each at 94°C for 15 seconds, annealing at 55°C for 30 seconds and extension at 72°C for 1 minute plus 5 seconds cycle elongation for each successive cycle, followed by a final extension at 72°C for 7 minutes. The amplification products were analyzed by staining with SYBR safe DNA gel stain, after electrophoresis on 1% agarose gel and then purified using Microcon YM-100 (Millipore Corporation, Bedford, MA). Then the purified amplification products and plasmid pIRES2-AcGFP1 were digested by restriction enzyme SalI and BamHI (10 U at 37°C for 120 minutes). These digested PCR products were purified from a 0.8% agarose gel using the S.N.A.P. Gel Purification Kit (Invitrogen, Paisley, UK), according to the manufacturer's protocol. The purified PCR products and plasmid pIRES2-AcGFP1 were analyzed on a second 0.8% agarose gel to confirm recovery. Finally, these purified PCR products were recombined into plasmid pIRES2-AcGFP1 using T4 DNA ligase kit (New England Biolabs, Ipswich, MA) and subjected transformation into *E. coli* DH5*α* (Invitrogen, Paisley, UK) according to manufacturer instruction. The successful construction was confirmed by PCR, BamHI, and Sal I digestion and DNA sequencing.

### Transfection of human sperm with plasmid pIRES2-AcGFP1-E6E7

Motile sperm were harvested by the swim-up technique as described above. The swim-up sperm fraction was divided in four samples: one used for the transfection with plasmid pIRES2-AcGFP1-E6E7, the second one used for the transfection with 2 µL Lipofectamine 2000 alone (Invitrogen, Paisley, UK), the third one used for the transfection with plasmid pIRES2-AcGFP1 and the last one as negative control.

Sperm were incubated in BWW supplemented with HEPES (10 mM, pH 7.4) and 3.5 mg/ml HSA (Human Serum Albumin) for capacitation at 37°C and 5% CO_2_ for 4 hours. Three hours after the beginning of capacitation, sperm were exposed to plasmid pIRES2-AcGFP1-E6E7. Briefly, a total of 100 µL mixture containing 800 ng plasmid pIRES2-AcGFP1-E6E7 and 2 μL Lipofectamine 2000 (Invitrogen, Paisley, UK) in BWW was incubated at room temperature for 20 minutes, and then was added to sperm sample and kept in the incubator for another 1 hour. After that, the sperm sample was washed at least five times in 5 mL of fresh BWW.

The successful of human sperm transfection with plasmid pIRES2-AcGFP1-E6E7 was confirmed by PCR. The DNA was extracted from sperm by QIAamp DNA Mini Kit (Qiagen, Valencia, CA), according to the manufacturer's protocol. Plasmid pIRES2-AcGFP1-E6E7 and sterile ddH_2_O were used as the positive and negative controls, respectively. The PCR mixture consisted of 5 µL 10× TaqGold buffer, 3 µL of MgCl_2_ 25 mM, 5 µL of each deoxynucleosidetriphosphate 200 µmol/L, 20 pmol of each primer (forward CMV: 5′-CGCAAATGGGCGGTAGGCGTG-3′ and reverse IRES: 5′-CCTCACATTGCCAAAAGACG-3′), 2.5 U TaqGold polymerase and sterile H_2_O in a final volume of 50 µL. The amplification program was as follows: denaturing at 94°C for 10 minutes, 37 cycles each at 94°C for 1 minute, annealing at 55°C for 1 minute and extension at 72°C for 2 minutes, followed by a final extension at 72°C for 10 minutes. The amplification products were analyzed by staining with SYBR safe DNA gel stain, after electrophoresis on 1% agarose gel.

### Hamster Egg Penetration Test (HEPT) with human sperm transfected with HPV E6/E7 plasmid

The HEPT was performed according to the World Health Organization [35] criteria with the use of commercially available frozen hamster eggs (Charles Rivers Laboratories, Wilmington, MA, USA) with human sperm transfected or not with plasmid pIRES2-AcGFP1-E6E7. After thawing, 120 eggs were washed three times in BWW and transferred to 0.1% trypsin to remove the zona pellucida. The zona-free hamster eggs were washed three times in BWW. Then, 15 eggs were placed in 100 µL containing 10^6^ motile spermatozoa under mineral oil. After 3 hours incubation at 37°C in a 5% CO_2_ atmosphere oocytes were recovered from the droplets and washed to loose adherent spermatozoa. To evaluate the successful sperm penetration, 40 oocytes (10 for each condition) were analyzed with the nuclear acid staining SYBRGreen (Invitrogen, Paisley, UK) under phase contrast microscopy and under the fluorescence microscope at 400× magnifications (Leica DMLB, Wetzlar, Germany). Sperm egg penetration test was expressed as mean number of sperm penetrated for oocyte (HSA). Twenty-four hours after insemination, the remaining oocytes were investigated under the fluorescence microscope (Leica DMLB, Wetzlar, Germany) at 400× magnifications. The oocytes were collected and classified into two groups: oocytes with green fluorescence and oocytes without green fluorescence. The oocytes from two groups were washed three times in cold 1× PBS to remove serum from the medium and then each oocyte was transferred singly into a PCR tube for PCR and RT-PCR.

### Single-Oocyte PCR for E6E7 genes

5 μL of cell lysis buffer were added to each oocyte, mixed then incubated at 65°C for 10 minutes. The cell lysate of oocyte was used as a DNA template. Plasmid pIRES2-AcGFP1-E6E7 and sterile ddH_2_O as the positive and negative controls, respectively. The first round multiplex PCR was performed with mixture consisted of 5 µL 10× TaqGold buffer, 3 µL of MgCl_2_ 25 mM, 5 µL of each deoxynucleosidetriphosphate 200 µmol/L, 20 pmol of each primer (forward CMV: 5′-CGCAAATGGGCGGTAGGCGTG-3′ and reverse IRES: 5′-CCTCACATTGCCAAAAGACG-3′), 2.5 U TaqGold polymerase and sterile H_2_O in a final volume of 50 μL. The amplification program was as follows: denaturing at 94°C for 10 minutes, 37 cycles each at 94°C for 1 minute, annealing at 55°C for 1 minute and extension at 72°C for 2 minutes, followed by a final extension at 72°C for 10 minutes. This first round multiplex PCR is followed by a nested PCR reaction with one of the same outer primer (forward CMV) combined with a unique inner primer (reverse: 5′-GCATAAATCCCGAAAAGCAA-3′). The amplification products were routinely analyzed by staining with SYBR safe DNA gel stain, after electrophoresis on 1,5% agarose gel.

### Single-Oocyte RT-PCR for E6/E7 expression

RNA extraction from each oocyte followed by RT-PCR was performed using the cells-to-cDNA II kit (Ambion, Austin, Texas). 10 µL of cold cell lysis buffer II were added to each oocyte, and incubated at 75°C for 10 minutes. 0.12 U DNase I was added to the mixture at 37°C for 30 minutes. Reverse transcription was preformed according to the kit protocol. PCR amplification was carried out with 10 µL cDNA of each sample from reverse transcription reaction as a template, E6/E7 specific primer pair (forward: 5′-GTCGACGTGATGCACCAAAAGAGAACTG-3′ and reverse: 5′- GCATAAATCCCGAAAAGCAA-3′) and two negative controls (no template and no reverse transcription). The amplification step was carried out using these conditions: denaturing at 94°C for 10 minutes, 37 cycles each at 94°C for 1 minute, annealing at 55°C for 1 minute and extension at 72°C for 1 minute, followed by a final extension at 72°C for 10 minutes. About 20 μL of each PCR product were made visible by staining with SYBR safe after electrophoresis on 1.5% agarose gel. This experiment was repeated three times under the same condition.

### Immunofluorescence of oocytes after penetration of human sperm incubated with HPV16-L1

The sperm were incubated or not with HPV16-L1 20 µg/ml and then were selected by swim-up for 45 minutes at 37°C, capacitated for 3 hours, and washed in BWW. Both samples were resuspended in fresh medium and the concentration of motile sperm was adjusted to 5 mil/mL. Meanwhile, the oocytes were prepared as described before. Fifteen eggs were placed in 100 µL containing 10^6^ motile sperm under mineral oil. After 3 hours of incubation at 37°C in a 5% CO_2_ atmosphere, oocytes were recovered from the droplets, washed free of loosely adherent spermatozoa. To perform immunofluorescence for HPV16-L1 in penetrated oocytes, samples were washed three times in BWW, allowed to air dry, fixed in PBS/paraformaldehyde 4% for 15 min and permeabilized with 3% PBS/Triton ×100 for 5 min (Sigma Aldrich, St. Louis, MO) at room temperature. All oocyte were then incubated with mouse monoclonal antibody HPV 16-L1 (CAMVIR-1) (0.8 µg/mL) (1∶250, Santacruz, Santa Cruz, CA) for 120 minutes at room temperature. Immunoreaction was then detected by sequential incubation with FITC-anti-IgG mouse secondary antibody (1∶200 from Bio-Rad, Berkeley, CA) for 60 minutes at room temperature. The slides were washed twice in PBS, and nuclei were counterstained with DAPI (5 mg/mL). Slides were mounted with anti-fade buffer and 24×24 mm coverslip. Finally, the oocytes were placed on to the slides and the immunostaining was evaluated with Nikon ViCo Video Confocal Microscope.
